# Bilateral Lung Injury with Delayed Pneumothorax following Preoperative Cryoanalgesia for Pectus Excavatum Repair in a 13-year-old Boy

**DOI:** 10.1055/a-2349-9668

**Published:** 2024-07-26

**Authors:** Clara Massaguer, Laura Saura-García, Pedro Palazón, Gastón Echaniz, Maria Carme Roqueta Alcaraz, Xavier Tarrado

**Affiliations:** 1 Department of Pediatric Surgery, Hospital Sant Joan de Deu, Universitat de Barcelona, Barcelona, Catalunya, Spain; 2 Department of Anesthesiology, Hospital Sant Joan de Déu Barcelona, Catalunya, Spain

**Keywords:** pneumothorax, cryoanalgesia, pectus excavatum, Nuss procedure, Minimally Invasive Pectus Excavatum Repair (MIRPE)

## Abstract

A 13-year-old male patient with marfanoid features and pectus excavatum with Haller index 4 and correction index of 38% underwent the Nuss procedure with cryoanalgesia 9 days prior, which transpired uneventfully. Preoperative spirometry was normal, and echocardiogram showed light aortic valve dilation. A month later, during a routine outpatient checkup, he referred middle abdominal pain, denying respiratory symptoms nor thoracic pain. He presented bilateral apical and right basal hypophonesis. Chest X-ray revealed bilateral pneumothorax and right pleural effusion. Consequently, the patient was admitted to the emergency room, and a chest computed tomography was ordered, reporting right apical blebs. Bilateral thoracoscopy was performed, and apexes were checked for pulmonary blebs to rule out primary pneumothorax. In the right chest, a wedge resection of a distorted area on the apex and pleuroabrasion were done. Four air leaking eschars were found when performing lung expansion under water as leaking test, corresponding to cryoanalgesia intercostal eschars, and subsequently closed by primary suture. In the left chest, there were no blebs. However, another four pleural lesions with intact pleura in the left lower lobe were also found. Postoperative course was uneventful and chest drains were removed 48 hours after surgery. He remains asymptomatic 21 months after discharge.

Cryoanalgesia in pectus excavatum is spreading due to the improvement in postoperative pain control. However, some complications may occur.

## Introduction

The repair of pectus excavatum (PE) with Nuss technique is a painful procedure. 1 2 3 4 In the last years, cryoanalgesia is spreading as it has shown benefits in postoperative pain management reducing the need of opioids. 5 6 7 8 9 10 11 Intercostal nerves' cryoneurolysis produces axonotmesis and initiates Wallerian degeneration distally to the site of nerve injury, resulting in temporary and reversible regional analgesia during weeks to months. 12 13 Cold temperatures are applied to the targeted nerves, commonly from −70 to −90°C in the case of PE, causing a transitory and reversible axonal disruption that inhibits afferent signal to the brain and thus reducing the pain in the treated area. It can either be applied during the Nuss procedure, 7 8 9 10 11 which increases operative time but enables direct thoracoscopic visualization (either unilateral or bilateral) and under only one general anesthesia, or ultrasound-guided a few days before surgery. 14 15 16 17 This second option ensures better analgesic control, as it takes from 6 hours up to 2 days to make maximum effect but requires two general anesthesia and ultrasound-guided puncture expertise. So far, the most commonly reported complications of this procedure include neuropathic pain, prolonged regional numbness, wound infection, and pneumothorax. 9 18 19 To the best of our knowledge, we report the first case of delayed pneumothorax caused by cryoanalgesia.

## Case Report

**Fig. 1:**
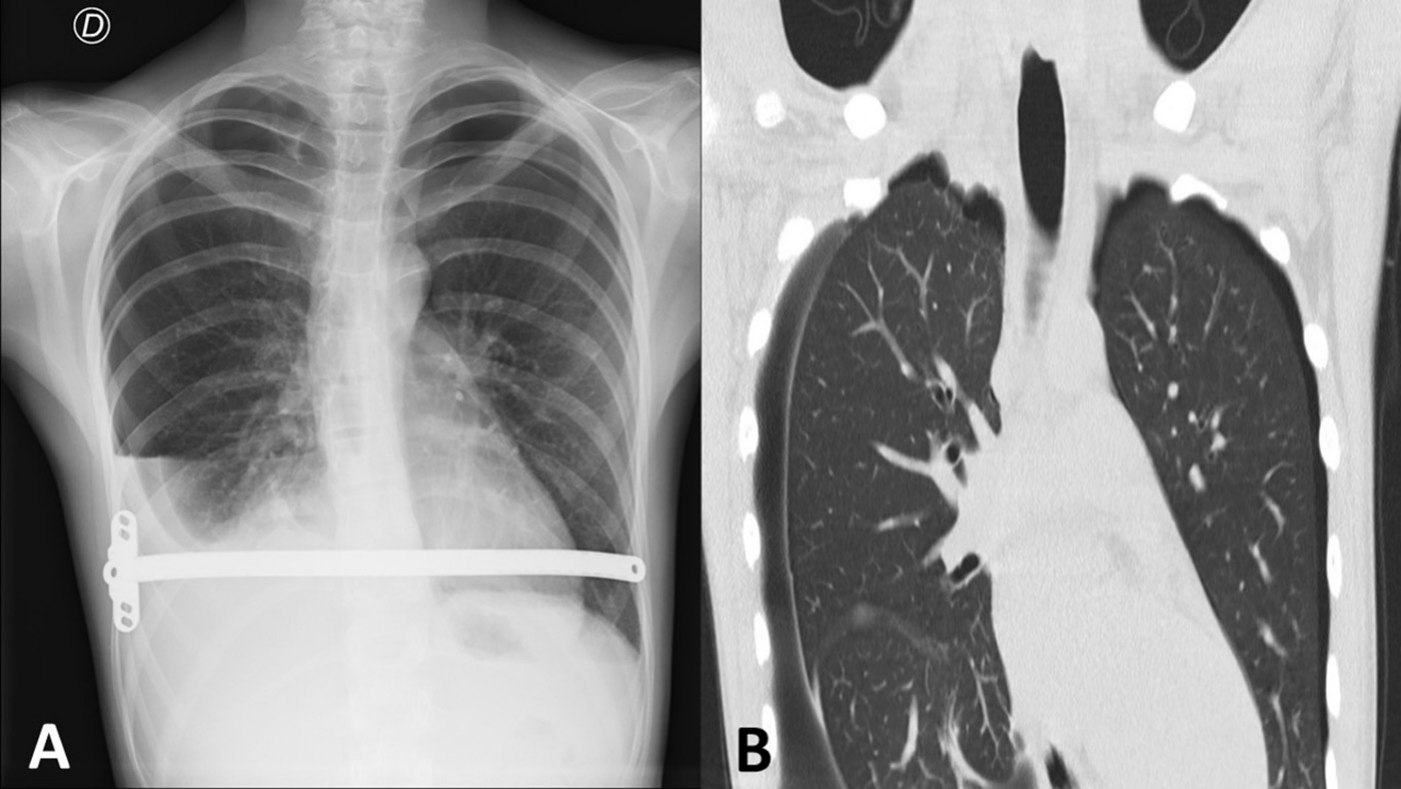
( **A** ) Chest X-ray displaying bilateral pneumothorax and right pleural effusion. ( **B** ) Chest computed tomography presenting right apical blebs.

**Fig. 2:**
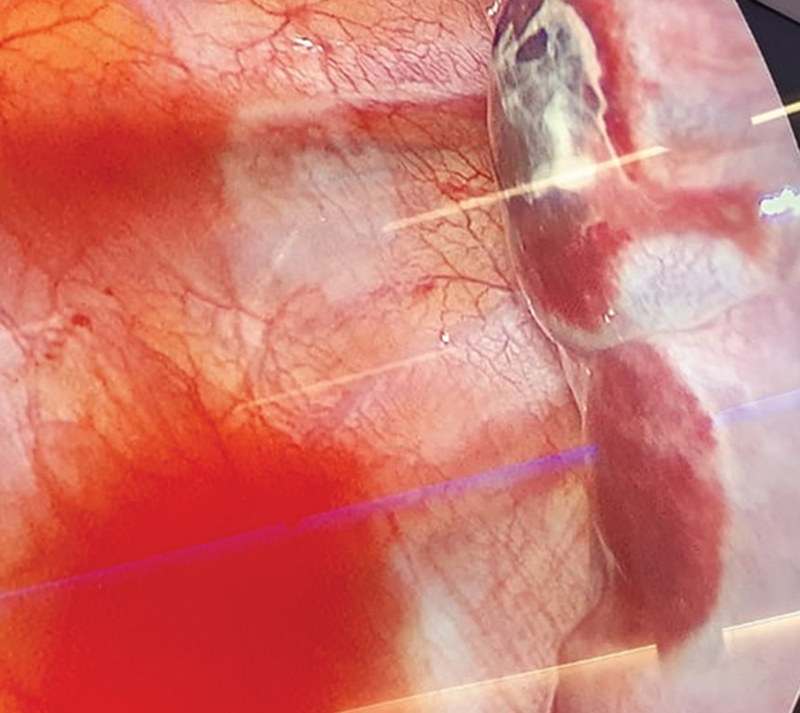
Thoracoscopic view revealing lung eschars secondary to cryoanalgesia. The right portion of the image displays the lung surface, with two rounded eschars that showed air leakage and were sutured.

A 13-year-old male patient with marfanoid features and PE (Haller index 4 and correction index of 38%), weighing 56 kg, standing 180 cm tall, underwent the Nuss procedure. In preoperative checkup, spirometry was normal and light aortic valve dilation was observed in echocardiogram. Also, preoperative genetic analysis for collagenopathies and overgrowth syndromes was normal. Ultrasound-guided percutaneous cryoanalgesia was performed 9 days before surgery in an outpatient basis, uneventfully. Under general anesthesia, 10 percutaneous punctures were performed, injecting normal saline in order to gently push the pleura forward and posteriorly applying a temperature of −88°C during a 2-minute cycle with the cryoprobe. The minimally invasive repair of PE was performed uneventfully, and pleural drainage was left for 5 days. The patient was discharged with bilateral full lung expansion. A month later, in routine outpatient checkup, he referred middle abdominal pain, denying respiratory symptoms or thoracic pain (although shortness of breath was noticeable). At physical exam, he presented bilateral apical and right basal hypophonesis. A chest X-ray was performed that showed bilateral pneumothorax and right pleural effusion ( Fig. 1 ). He was admitted to the emergency room and chest computed tomography was ordered, reporting right apical blebs ( Fig. 1 ). With the suspicion of primary bilateral simultaneous pneumothorax, bilateral thoracoscopy was performed. Apexes were checked for pulmonary blebs to rule out spontaneous pneumothorax. In the right chest, a wedge resection of a distorted area on the apex and pleuroabrasion were done. Four air-leaking eschars, corresponding to cryoanalgesia intercostal eschars, were found in the posterior surface of the right lower lobe when performing lung expansion under water as leaking test ( Fig. 2 ). They were closed by primary suture with a 3–0 absorbable braided material. In the left chest, there were no blebs and another four more superficial lesions with no pleural discontinuity in the left lower lobe were also found. As they did not present air leakage, they were not sutured. Bilateral chest drains were placed, which were removed at 48 hours. Postoperative course was uneventful. He remains asymptomatic 21 months after discharge.

## Discussion

At our institution, cryoneurolysis protocol in PE patients was established in October 2021. Ultrasound-guided percutaneous cryoanalgesia is performed on an outpatient basis a few days prior to surgery to ensure a proper analgesic effect. 12

**Fig. 3:**
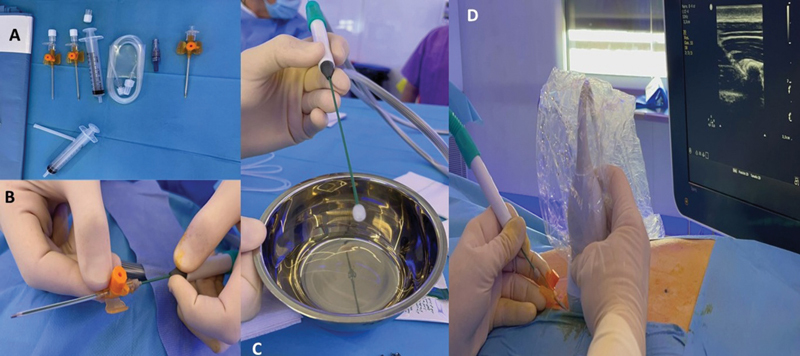
Ultrasound-guided percutaneous cryoanalgesia procedure. ( **A** ) Material currently used in the procedure. ( **B** ) Marking of the probe with adhesive tape. ( **C** ) Ice ball created with the cryoprobe. ( **D** ) Ultrasound-guided percutaneous puncture observing a round hypoechogenic image corresponding to the ice ball.

After this case, we examined for lung eschars in the successive procedures, noticing that another patient had the same lesions but more superficial, without air leakage as the visceral pleura was intact. Therefore, the technique and material was modified to avoid lung damage. Nowadays, cryoneurolysis is applied in 10 intercostal spaces: 5 in every side, starting at the level of the deepest chest depression, from two spaces above to two spaces below, which are marked with a surgical marker. The probe is introduced through a 14-gauge beveled intravenous catheter that serves as a guide-through. To make sure the probe will not go too deep, a mark made with adhesive skin closure is placed on the probe at the point where only its tip overtakes the catheter ( Fig. 3 ). Normal saline is still inyected to verify the needle tip placement and to push the pleura forward. We aim to place the probe's tip between intern and innermost intercostal muscles and avoid going underneath the rib. Ultrasound-guided percutaneous puncture is done, leaving the catheter in place and replacing the needle with the probe ( Fig. 3 ). Freezing is applied for 2 minutes at −88°C. Color Doppler twinkling artifact to delineate the probe's contour and hypoechoic image corresponding to the ice ball are ultrasound signs that help locate the probe's position. The same process is repeated for each intercostal space. While the cryoprobe is performing the cycle, the next intercostal space is punctured with the intravenous catheter to optimize time. Once the cycle is finished, the catheter is already placed and ready to introduce the cryoprobe through it. At the end of the procedure, a lung ultrasound and a chest X-ray are performed to rule out immediate complications. Ever since the material and technique was modified by puncturing more superficially with the angiocath and marking the probe; no more lung eschars have been observed.

## Conclusion

The use of cryoanalgesia in PE is increasing due to the improvement in postoperative pain control management. However, some complications may occur. Both the anesthesiologist and the pediatric surgeon must exercise caution to ensure the proper device and technique are used to avoid potential complications. Percutaneous intercostal cryoanalgesia can cause delayed pneumothorax due to lung eschars. To the best of our knowledge, this is the first reported direct visualization of cryoanalgesia lung injuries causing delayed pneumothorax. Regarding this complication, adjustments were made to the ultrasound-guided percutaneous cryoanalgesia technique, employing a more superficial approach. In other words, avoiding too-close application of the cryoprobe to the lung surface can overcome this complication. Thus far, we have not observed any additional occurrences of lung lesions ( Supplementary Fig. S1 , available in the online version).

## Notes

Importance for a Pediatric Surgeon Cryoanalgesia may cause lung injuries by contact leading to pneumothorax. Anesthesiologists and pediatric surgeons should be aware of this complication and avoid too deep ultrasound-guided percutaneous cryoneurolysis. Injecting normal saline in order to gently push the pleura forward and the placement of a mark on the probe may be helpful to prevent this complication.

## References

[1] Nuss D Obermeyer R J Kelly R E Nuss bar procedure: past, present and future Ann Cardiothorac Surg 2016 5 05 422 433 27747175 10.21037/acs.2016.08.05 PMC5056934

[2] Ma B Sun Y Hao C Liu X Shen S Patient-controlled intravenous analgesia with or without ultrasound-guided bilateral intercostal nerve blocks in children undergoing the Nuss procedure: a randomized, double-blinded, controlled trial Pain Res Manag 2022 2022 5.776833E6 10.1155/2022/5776833 PMC9337970 35910406

[3] Frawley G Frawley J Crameri J A review of anesthetic techniques and outcomes following minimally invasive repair of pectus excavatum (Nuss procedure) Paediatr Anaesth 2016 26 11 1082 1090 27510834 10.1111/pan.12988

[4] Kirupaharan S Briatico D Robinson T Fitzgerald P Walton J M Postoperative management of pediatric patients undergoing minimally invasive repair of pectus excavatum: where are we now? J Pediatr Surg 2022 57 05 927 931 35058061 10.1016/j.jpedsurg.2021.12.049

[5] Lai K Notrica D M McMahon L E Cryoablation in 350 Nuss procedures: evolution of hospital length of stay and opioid use J Pediatr Surg 2023 58 08 1435 1439 36494205 10.1016/j.jpedsurg.2022.10.051

[6] Arshad S A Ferguson D M Garcia E I Hebballi N B Buchanan A C Tsao K Cryoanalgesia is associated with decreased postoperative opioid use in minimally invasive repair of pectus excavatum J Surg Res 2022 271 1 6 34814047 10.1016/j.jss.2021.10.011

[7] Cockrell H C Hrachovec J Schnuck J Nchinda N Meehan J Implementation of a cryoablation-based pain management protocol for pectus excavatum J Pediatr Surg 2023 58 07 1239 1245 36894442 10.1016/j.jpedsurg.2023.01.059

[8] Keller B A Kabagambe S K Becker J C Intercostal nerve cryoablation versus thoracic epidural catheters for postoperative analgesia following pectus excavatum repair: preliminary outcomes in twenty-six cryoablation patients J Pediatr Surg 2016 51 12 2033 2038 27745867 10.1016/j.jpedsurg.2016.09.034

[9] Rettig R L Rudikoff A G Lo H YA Cryoablation is associated with shorter length of stay and reduced opioid use in pectus excavatum repair Pediatr Surg Int 2021 37 01 67 75 33210165 10.1007/s00383-020-04778-x

[10] Cadaval Gallardo C Martínez J Bellía-Munzon G Thoracoscopic cryoanalgesia: a new strategy for postoperative pain control in minimally invasive pectus excavatum repair. Crioanalgesia toracoscópica: nueva estrategia para el control del dolor postoperatorio en cirugía del pectus excavatum Cir Pediáatr 2020 33 01 11 15 32166917

[11] Morikawa N Laferriere N Koo S Johnson S Woo R Puapong D Cryoanalgesia in patients undergoing Nuss repair of pectus excavatum: technique modification and early results J Laparoendosc Adv Surg Tech A 2018 28 09 1148 1151 29672193 10.1089/lap.2017.0665

[12] Whittaker D K Degeneration and regeneration of nerves following cryosurgery Br J Exp Pathol 1974 55 06 595 600 4447794 PMC2072730

[13] Evans P J Cryoanalgesia. The application of low temperatures to nerves to produce anaesthesia or analgesia Anaesthesia 1981 36 11 1003 1013 7316118 10.1111/j.1365-2044.1981.tb08673.x

[14] Djebbar S Rossi I M Adler R S Ultrasound-guided cryoanalgesia of peripheral nerve lesions Semin Musculoskelet Radiol 2016 20 05 461 471 28002868 10.1055/s-0036-1596063

[15] Byas-Smith M G Gulati A Ultrasound-guided intercostal nerve cryoablation Anesth Analg 2006 103 04 1033 1035 17000825 10.1213/01.ane.0000237290.68166.c2

[16] Nicolás M Acosta C M Martinez Ferro M Short communication: ultrasound-guided percutaneous cryoanalgesia of intercostal nerves for uniportal video-assisted thoracic surgery Ultrasound J 2022 14 01 33 35907076 10.1186/s13089-022-00284-4 PMC9339062

[17] Finneran Iv J J Gabriel R A Swisher M W Ultrasound-guided percutaneous intercostal nerve cryoneurolysis for analgesia following traumatic rib fracture -a case series Korean J Anesthesiol 2020 73 05 455 459 31684715 10.4097/kja.19395 PMC7533180

[18] Zobel M J Ewbank C Mora R Idowu O Kim S Padilla B E The incidence of neuropathic pain after intercostal cryoablation during the Nuss procedure Pediatr Surg Int 2020 36 03 317 324 31760443 10.1007/s00383-019-04602-1

[19] Parrado R Lee J McMahon L E The use of cryoanalgesia in minimally invasive repair of pectus excavatum: lessons learned J Laparoendosc Adv Surg Tech A 2019 29 10 1244 1251 31259649 10.1089/lap.2019.0203

