# Supplementary material for: Bilateral Lung Injury with Delayed Pneumothorax following Preoperative Cryoanalgesia for Pectus Excavatum Repair in a 13-year-old Boy
Source: European J Pediatr Surg Rep. 2024 Jul 26;12(1):e50–3. doi: 10.1055/a-2349-9668 (PMC11281861; doi:10.1055/a-2349-9668)
Supplement: Supplementary file 1 — Supplementary Material [file 10-1055-a-2349-9668-s2023050704cr.pdf]

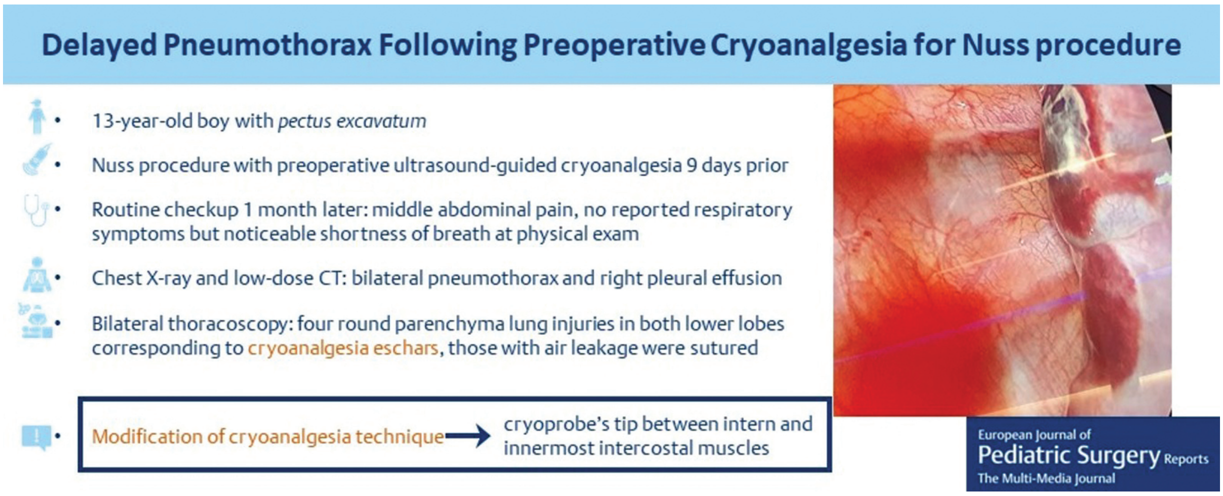

Supplementary Fig. S1 Case report of a delayed pneumothorax caused by preoperative cryoanalgesia for Nuss procedure. The figure shows a summary of the case and implemented actions.

 © 2024. The Author(s).
